# Evaluating the Effectiveness of Hospital Antiseptics on Multidrug-Resistant *Acinetobacter baumannii*: Understanding the Relationship between Microbicide and Antibiotic Resistance

**DOI:** 10.3390/antibiotics11050614

**Published:** 2022-05-03

**Authors:** Melanie Betchen, Holly M. Giovinco, Michael Curry, Jackson Luu, Henry Fraimow, Valerie J. Carabetta, Raquel Nahra

**Affiliations:** 1 Department of Internal Medicine, Cooper University Hospital, Camden, NJ 08103, USA; betchen-melanie@cooperhealth.edu; 2 Department of Biomedical Sciences, Cooper Medical School of Rowan University, Camden, NJ 08103, USA; giovin24@rowan.edu (H.M.G.); currym75@rowan.edu (M.C.); jl2190@scarletmail.rutgers.edu (J.L.); 3 Division of Infectious Diseases, Department of Medicine, Cooper University Hospital, Camden, NJ 08103, USA; fraimow-henry@cooperhealth.edu

**Keywords:** bacteria, multidrug resistance, microbicide resistance, biocide, antiseptic, germicide, hospital-acquired infections, efflux pumps

## Abstract

*Acinetobacter baumannii* hospital infections are difficult to treat due to the rapid emergence of multidrug-resistant (MDR) strains. In addition, *A. baumannii* can survive in numerous adverse environments, including in the presence of common hospital antiseptics. We hypothesized that in addition to accumulating drug resistance determinants, MDR *A. baumannii* strains also accumulate mutations that allow for greater microbicide tolerance when compared to pan-susceptible (PS) strains. To test this hypothesis, we compared the survival of five MDR and five PS patient isolates when exposed to bleach, ethanol, quaternary ammonium compounds, chlorhexidine gluconate, and povidone. We evaluated bacteria in a free-living planktonic state and under biofilm conditions. Each disinfectant eliminated 99.9% of planktonic bacteria, but this was not the case for bacterial biofilms. Next, we characterized strains for the presence of the known microbicide-resistance genes *cepA*, *qacEΔ1*, *qacE*, and *qacA*. MDR strains did not survive more than PS strains in the presence of microbicides, but microbicide-resistant strains had higher survival rates under some conditions. Interestingly, the PS strains were more likely to possess microbicide-resistance genes. Microbicide resistance remains an important topic in healthcare and may be independent of antimicrobial resistance. Hospitals should consider stricter isolation precautions that take pan-susceptible strains into account.

## 1. Introduction

Hospital-acquired infections (HAIs), especially those from Gram-negative organisms that tend to become multidrug resistant (MDR), pose serious threats to healthcare systems [1]. *Acinetobacter baumannii* is a non-spore forming, Gram-negative rod that is ubiquitous in the environment, and an opportunistic human pathogen. *A. baumannii* infections commonly occur in severely ill patients with extended hospital stays. These bacteria readily acquire antibiotic resistance, resulting in difficult to treat infections which may require higher doses and longer treatment durations of more toxic antibiotics [2,3,4,5]. *A. baumannii* is one of the five pathogens labeled as an urgent threat by the Centers for Disease Control and Prevention (CDC), due to its ability to become resistant to multiple standard-of-care antibiotics [6]. In fact, roughly 50% of *A. baumannii* isolates are MDR [7]. The mechanisms of resistance found in this bacterium include the presence of beta lactamases, mutation of target sites, changes in membrane permeability, and overproduction of efflux pumps [8]. First-line treatments for *A. baumannii* infections typically include cefepime, ampicillin-sulbactam, or meropenem [7]. However, with the emergence of carbapenem-resistant pathogens, newer agents such as cefiderocol, a novel beta-lactam antibiotic that uses a siderophore moiety to bind to bacterial iron transporters, have been used [9,10]. Cefiderocol was shown to be as effective as imipenem-cilastatin for urinary tract infections in a clinical trial [11], but was associated with higher mortality rates compared to other standard treatments in another study [10]. Given this, treatment options for MDR *A. baumannii* remain limited.

Most infections (greater than 90%) are nosocomial rather than community acquired [12]. Pathogens are classified as urgent, serious, or concerning threats according to the CDC and are monitored through the Multi-site Gram-negative Surveillance Initiative [13]. Carbapenem-resistant *Acinetobacter baumannii* (CRAB) and carbapenem-resistant *Enterobacterales* (CRE) are considered urgent threats, while other highly resistant organisms, such as vancomycin-resistant *Enterococci* (VRE) and multidrug-resistant *Pseudomonas aeruginosa*, are considered serious threats. Compared to other multidrug-resistant organisms, the number of CRAB infections tends to be lower, but still substantial. In 2017, there were 8500 hospital infections from CRAB that resulted in over 700 deaths, compared to 13,100 hospital infections with 1100 deaths from CRE in the United States. In the same year, there were 54,500 hospital infections with 5400 deaths from MDR *P. aeruginosa* and 32,600 hospital infections with 2700 deaths from VRE [14].

Globally, there were approximately 1,000,000 *A. baumannii* HAIs in 2019, about half of which were resistant to carbapenems [15]. The presence of multidrug resistance varies by country. A 2019 metanalysis evaluated the prevalence of *A. baumannii* hospital and ventilator associated pneumonia (HAP and VAP) and found that Central America, Cuba, Mexico, and Latin America/Caribbean had a lower rate of infections, but 100% of those infections were MDR [16], compared to the United States that had nearly triple the amount of HAPs and VAPs with an MDR prevalence of 69% [16]. During that same year, the prevalence of HAP and VAP infections from MDR *A. baumannii* was 91% in Greece [16], and a 4-year cohort study conducted in Greece showed that the all-cause hospital mortality rate among 91 patients with a pandrug-resistant (PDR) *A. baumannii* infection was as high as 68% [17]. Another study conducted on 92 Tunisian ICU patients showed a mortality rate up to 60% among those infected with *A. baumannii* ventilator associated pneumonia [18]. Furthermore, an analysis of 1423 U.S. patients with *A. baumannii* infections from 2009 to 2013 revealed that 80% were MDR [19]. Patients infected with MDR strains typically have more severe illness and are associated with higher frequencies of inappropriate antibiotic therapy, further perpetuating the rise of MDR isolates [19].

MDR *A. baumannii* infections are difficult to treat, which makes controlling hospital outbreaks extremely important, yet it remains a difficult task. This is largely due to *A. baumannii’s* ability to survive in many different environments and under adverse conditions [20,21,22,23,24]. They have evolved different survival mechanisms, such as resistance to desiccation [12], which allows for survival for extended periods of time on surfaces [4,12]. In fact, one study revealed the presence of *A. baumannii* on the bedrails of a previously infected patient up to 9 days after discharge [12,25]. This emphasizes the importance of proper decontamination of hospital rooms in preventing the spread of MDR bacteria. Germicides and microbicides are synonymous terms defined by the CDC as agents that destroy microorganisms [26]. Microbicides are routinely used in the hospital environment for disinfection and sterilization to limit the spread of nosocomial infections. *A. baumannii* has an innate ability to become resistant to microbicides, further contributing to its ability to survive in the harshest conditions [27]. For example, in 283 *A. baumannii* ICU isolates, the minimum inhibitory concentrations (MICs) increased after treatment with benzalkonium chloride (BAC) and chlorhexidine gluconate (CHG). In addition, the minimum bactericidal concentrations (MBCs) for drug-resistant isolates were also generally higher [27]. Biofilm formation enhances microbicide resistance. *A. baumannii* biofilms are more resistant than planktonic bacteria to BAC and CHG on ceramic surfaces [28]. Compounding the problem further, *Acinetobacter* strains that form biofilms are frequently multidrug resistant [29]. As biofilms are also inherently more drug-tolerant, some newer technologies are being explored as alternative therapeutics, including silver nano-particles [30]. In a promising study, nanoparticles were shown to reduce biofilm formation and downregulate the expression of biofilm producing genes among *A. baumannii* wound isolates [31]. However, evaluation of safety and efficacy is still underway [30]. *Acinetobacter* biofilms remain challenging to eradicate from surfaces and such infections are still difficult to treat.

The mechanism of action for each antiseptic agent varies. The mechanism of action for ethanol, CHG, and quaternary ammonium compounds (QACs) is the degradation of cell membranes [32], resulting in cell lysis and further leading to the degradation of cellular proteins and enzymes [33]. Bleach is a potent oxidizer and disrupts cellular activities, such as protein synthesis. The skin antiseptic povidone rapidly degrades proteins [32]. Resistance may emerge when an agent is used at a sub-lethal concentration, or for an inappropriate amount of time [34]. The overproduction of certain efflux pumps is a mechanism of microbicide resistance employed by bacterial pathogens [33]. The QAC-specific efflux pumps are members of the small multidrug-resistance (SMR) family [35], and many of the mechanisms that lead to antibiotic resistance, coincide with the mechanisms of microbicide resistance [5,34,36]. For example, the *qacE* efflux pump gene is commonly found on integrons with the *sul1* gene, which confers resistance to sulfonamides [35]. Furthermore, the presence of *qacEΔ1* and *qacE* has been correlated with the presence of certain beta-lactamase genes (*bla*_VIM_ and *bla*_NDM-1_) and has additionally been associated with tolerance to CHG [5]. CepA is an efflux pump that increases the MICs of *Klebsiella pneumoniae* after exposure to CHG [32,37]. While the *qacA* gene is frequently seen among Gram-positive pathogens [38], a recent study of 44 *A. baumannii* isolates in Saudi Arabia revealed that 16.7% of strains contained the *qacA* gene [39].

The role that microbicide-resistance genes play in hospital outbreaks remains controversial. One study reported that the *qacE* and *qacEΔ1* genes were identified among 100 MDR *A. baumannii* isolates from an ICU, however no evidence of microbicide tolerance was identified [40]. In agreement, a different study found no differences in bacterial survival among 20 MDR strains and 20 sporadic pan-susceptible strains after exposure to antiseptics [41]. Furthermore, there was no significant correlation between the presence of *cepA*, *qacE*, and *qacEΔ1* genes and an increased MIC among 44 MDR *A. baumannii*, *Pseudomonas aeruginosa*, and *K. pneumoniae* strains [39]. However, another study found that *A. baumannii* strains that were resistant to carbapenems, fluoroquinolones, aminoglycosides, and tetracyclines were associated with microbicide tolerance [42].

Hospitals routinely use a variety of disinfecting agents, such as QACs, bleach, and ethanol. These are usually in the form of hospital disinfectant wipes and are used to clean surfaces and medical equipment. Antiseptics such as 10% povidone and 2% CHG are also commonly used for hand hygiene. For antiseptics to be effective against bacteria they must be used at specific contact or dwell times and meet certain Food and Drug Administration (FDA) standards. In general, this requires the germicide to kill 99.9% of bacteria on a hard, non-porous surface after a maximum time of 5 min [43]. Different disinfectant wipes require different dwell times. Sani-cloth^®^ wipes containing bleach are considered bactericidal, viricidal, and fungicidal when a dwell time of 4 min is used. However, the efficacy against certain pathogens varies based on dwell time [44]. At 1 min, it is effective against *A. baumannii* isolates that are resistant to cefazolin, ampicillin, gentamicin, piperacillin, trimethoprim-sulfamethoxazole, and intermediately resistant to cefotaxime and ceftriaxone, but not necessarily carbapenem-resistant *A. baumannii* [44]. Furthermore, while it is effective against *A. baumannii* after 1 min, it is not effective against all pathogens until after 4 min of disinfection [44]. Ethanol typically has a dwell time of 30 s to 1 min [45]. QAC wipes have different dwell times depending on the components. 0.61% dodecyl dimethyl ammonium chloride in 27% ethanol and 25% isopropanol (0.61% DDAC) is used mainly in the ICU and has a dwell time of 1 min [46], whereas the combination QAC wipe of 0.25% alkyl dimethyl ethyl benzyl ammonium chloride, 0.25% alkyl dimethyl benzyl ammonium chloride in 55% isopropanol (0.50% BAC) has a dwell time of 2 min [47].

Most of the research performed so far on microbicide tolerance or resistance among *A. baumannii* isolates are performed under optimal settings with microbicides used at the proper dwell times. Therefore, a possible reason for hospital outbreaks of multidrug-resistant organisms could be due to improper cleaning techniques. Our goal is to explore the interaction of MDR bacteria with microbicides as potential contributors to hospital outbreaks. We hypothesized that in addition to accumulating drug-resistance determinants, MDR *A. baumannii* also accumulates mutations that allow for greater antiseptic tolerance when compared to pan-susceptible (PS) strains. We found no significant differences between MDR and PS strain survival among planktonic bacteria. As we did not find significant differences between MDR and PS strains under planktonic conditions, we tested the efficacy of antiseptic agents against bacterial biofilms. Our results demonstrated increased bacterial survival, but no differences between MDR and PS strains. Finally, we determined which strains contained the known microbicide-resistance genes and showed that significantly more microbicide-resistant bacteria survived compared to microbicide-sensitive bacteria after specific treatments. Interestingly, we found that the presence of microbicide-resistance genes was more likely in PS strains. This suggests that PS *A. baumannii* may be underestimated in the propagation of hospital outbreaks and highlights the need for increased hospital precautions for dealing with the spread of both MDR and PS organisms.

## 2. Results

### 2.1. Antiseptic Tolerance of Planktonic Acinetobacter baumannii Isolates

**Figure 1:**
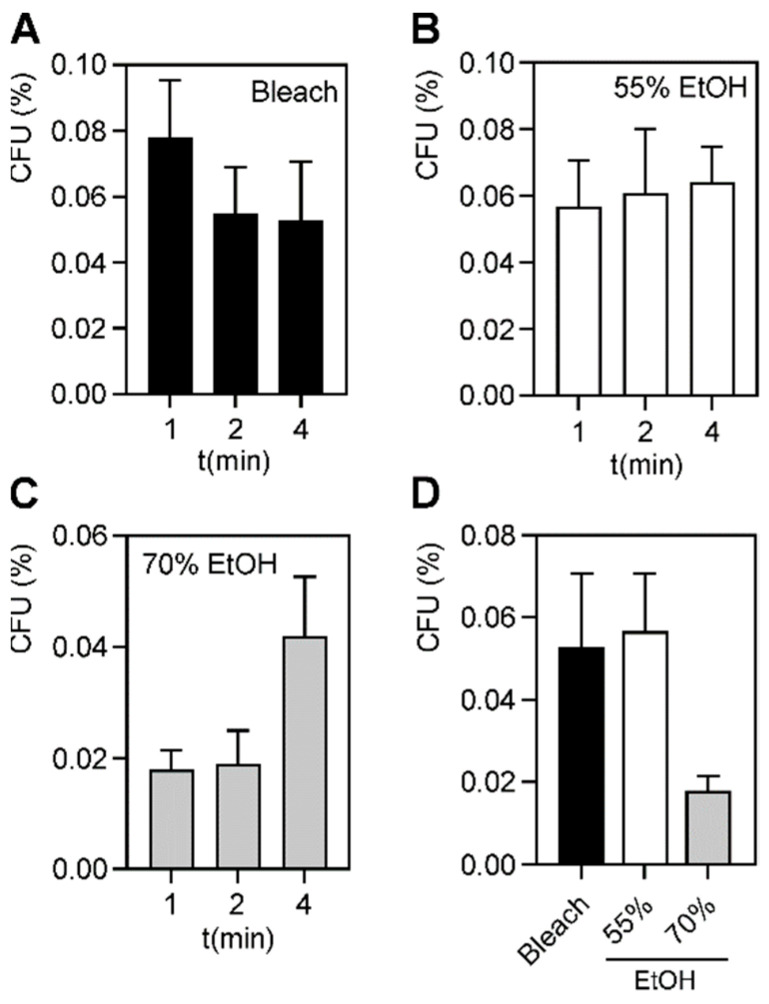
Survival of planktonic bacteria following germicide exposure. Average percent survival of ten planktonic *A. baumannii* strains, both MDR and PS strains combined, after 1-, 2-, and 4-min exposure with (**A**) 0.63% bleach, (**B**) 55% ethanol, and (**C**) 70% ethanol. (**D**) Comparison of bacterial survival at proper contact times for bleach (4 min), and 55% and 70% ethanol (1 min). A *p*-value of ≤0.05 was considered significant. The bars show mean survival among all bacterial strains, and error bars represent standard deviation. No significant differences were observed.

*A. baumannii* has evolved to survive and persist in harsh environments [4,12,20,21,22,23,24], including in the presence of common hospital antiseptics [5,42]. Hospital outbreaks of *A. baumannii* are becoming more common, especially among patients in ICUs. We hypothesized that MDR *A. baumannii* outbreaks occur because these strains are more tolerant or resistant to microbicides than susceptible strains. We thought it possible that some drug-resistance mechanisms may offer cross protection against some antiseptic agents. To test this idea, we performed time kill assays using five MDR and five PS *A. baumannii* patient isolates, in the presence and absence of various disinfectants and skin antiseptics. Free-living, planktonic *A. baumannii* strains were exposed to antiseptics for 1, 2 and 4 min, and survivors plated on rich media. Every antiseptic agent tested killed >99% of either MDR or PS bacteria. The most efficacious antiseptics were the QAC’s, and the skin antiseptics CHG and povidone, which completely eradicated all bacteria (Table S1). For 0.63% bleach, 55% and 70% ethanol, a small subpopulation (<1%) of bacteria survived treatment, even beyond the recommended dwell times (Figure 1A–C, Tables S1 and S2).

**Figure 2:**
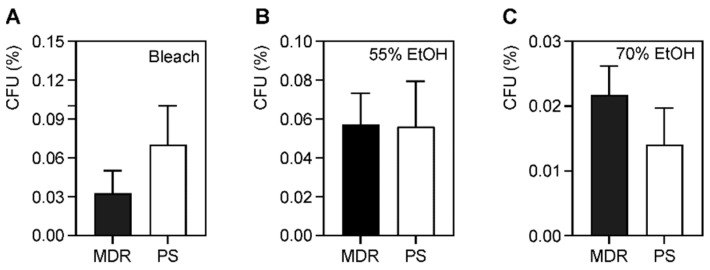
Comparison of survival of microbicide exposure of multidrug-resistant and pan-susceptible strains. Average percent survival of five MDR and five PS planktonic *A. baumannii* strains following exposure to (**A**) 4 min of bleach, (**B**) 1 min of 55% ethanol, and (**C**) 1 min of 70% ethanol. The bars show mean survival and error bars represent standard deviation. A *p*-value of ≤0.05 was considered significant. No significant differences were observed.

There were no statistical differences among the three treatments (Figure 1D). To determine if the MDR strains survived better than the PS strains, we performed unpaired *t*-tests among the two separate groups (Figure 2). Again, no statistical differences were observed between any of the three antiseptics, when used at their proper dwell times. Our results suggest that the drug-resistance properties of the bacterial strains do not directly confer increased survival in the presence of common hospital antiseptic agents (Figure 2 and Table S2).

### 2.2. The Presence of Known Microbicide Resistance Genes Confers Higher Tolerance, and Is more Prevalent among Susceptible Strains

Since we did not observe any differences between the survival of MDR and PS strains, we checked the strains for the presence of microbicide-resistance genes. Some known microbicide resistance genes have been characterized in *A. baumannii* [5]. To test for the presence of some common efflux pump genes, including *qacA*, *qacE*, *qacEΔ1* and *cepA*, we amplified each gene by PCR. All ten strains contained the *cepA* resistance gene, however only four isolates additionally contained both *qacE* and *qacEΔ1*. None of the isolates contained the *qacA* gene (Table 1).

**Figure 3:**
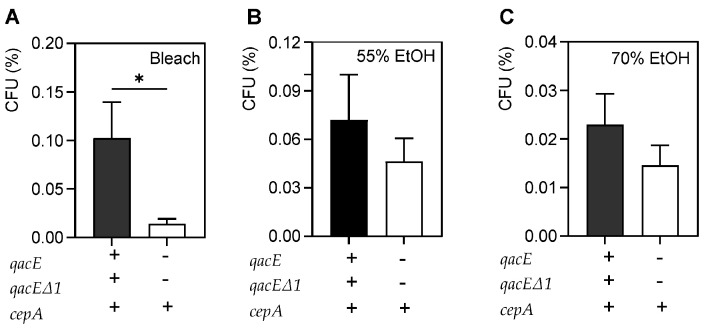
Comparison of survival of microbicide exposure of microbicide-resistant and -susceptible strains. Average percent survival of four microbicide-resistant, planktonic bacteria vs. six microbicide-sensitive strains after exposure to antiseptics at proper dwell times. (**A**) Microbicide-resistant strains survived more than microbicide-sensitive bacteria after 4 min of bleach exposure (*p* = 0.01). No differences were seen after 1 min of 55% ethanol exposure (**B**), or 1 min of 70% ethanol exposure (**C**). The bars show mean survival and error bars represent standard deviation. A *p*-value of ≤0.05 was considered significant. * *p*-value < 0.05.

We presumed that the presence of three microbicide-resistance genes would lead to more tolerance to microbicides if they were expressed. The strains were grouped as microbicide-sensitive, containing only *cepA*, and microbicide-resistant, which contained *qacE*, *qacEΔ1*, and *cepA*. Bacterial survival was evaluated based on microbicide resistance for each antiseptic using unpaired *t*-tests (Figure 3). Microbicide-resistant bacteria among planktonic bacteria survived more compared to microbicide-sensitive bacteria after treatment with bleach (*p* = 0.01) but not after 55% and 70% ethanol. This suggests that the presence of these efflux genes may confer microbicide tolerance to bleach, irrespective of their drug-resistance status.

### 2.3. Determination of MICs and MBCs of Components of Antiseptic Wipes

Although the MDR strains were not found to be more resistant to the clinical disinfecting agents than susceptible strains, it is possible that they have an increased MIC or MBC. To test this idea, we determined the MIC and MBC of the *A. baumannii* strains to each component of the hospital wipes, including bleach, ethanol, and isopropanol. MIC determination for the QACs was not possible, as at higher concentrations, a precipitate formed that led to an artificially high optical density (OD) reading when no bacterial growth was observed by plating. In general, we found that the hospital concentrations used were well above the MICs and MBCs for bleach, ethanol, and isopropanol (Table 2, Table 3 and Table 4).

As expected, the MBCs for all microbicides were higher than their respective MICs. Generally, an MBC/MIC ratio >4 indicates that the agent has bacteriostatic properties, while a ratio of <4 indicates the agent is bactericidal [48,49]. If looking at the average MBC/MIC ratio for all strains, bleach is bactericidal (Table 2). However, if the strains are separated by drug or microbicide-resistance properties, differences emerge. While the MICs for all groups are very similar, the MBCs for the MDR and microbicide-resistant strains were increased compared to the corresponding sensitive strains (Table 2). The MBC/MIC ratio becomes >4 after bleach treatment for MDR and microbicide-resistant strains, indicating bleach has bacteriostatic properties against these strains. This aligns with our prior time-kill assay results, which showed planktonic microbicide-resistant strains survived more after bleach exposure compared to microbicide-sensitive ones (Figure 3). The MBC for the MDR strains in ethanol was increased compared to the PS strains, however, ethanol was still bactericidal against these strains (Table 3). A similar observation was made for the microbicide-resistant strains with isopropanol (Table 4). The increase in MBC indicates a greater risk for development of microbicide resistance [41].

### 2.4. Antiseptic Tolerance of A. baumannii Biofilms

**Figure 4:**
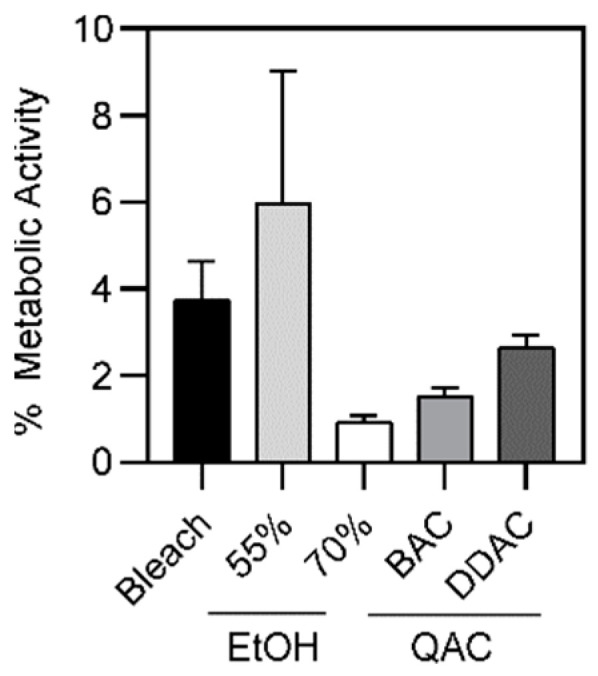
Bacterial biofilm survival when exposed to various microbicides. A comparison of average biofilm survival when exposed to various microbicidal agents. No significant differences were observed between all microbicides at their proper contact times (4 min for bleach, 1 min for 55% and 70% ethanol and DDAC, and 2 min for BAC). The average metabolic activity of all strains is displayed; error bars represent standard deviation.

Since we did not observe any differences among MDR and PS *A. baumannii* strains when grown planktonically, we next determined if bacteria that were present in biofilms would demonstrate any differences in microbicide survival. Biofilm formation in *A. baumannii* is an important virulence factor that allows for survival in adverse environments, including on polycarbonate, which is often used in the production of medical equipment [50,51,52]. Bacterial biofilms were formed following static growth in 96-well plates for 48 h. Non-adherent cells were removed, and resulting biofilms were exposed to antiseptic agents for 1, 2 and 4 min. Biofilm metabolic activity was determined using an XTT-reduction assay and used as a proxy to determine biofilm viability after exposure to microbicidal agents. Overall, there was significantly more survival among bacteria in biofilms compared to free living (compare Figure 4 to Figure 1D, Table S3). We found that there was 3.7% survival after 4 min of bleach exposure, 6.23% after 1 min of 55% ethanol, and 3.3% after 1 min of 70% ethanol. Whereas previously the QAC formulations led to complete killing of planktonic bacteria, in biofilms there was 2.6% survival after exposure to 1 min of DDAC, and 1.5% after 2 min of exposure of 0.5% BAC (Figure 4). Among the different antiseptic treatments, these differences were not significant (Figure 4 and Table S3).

**Figure 5:**
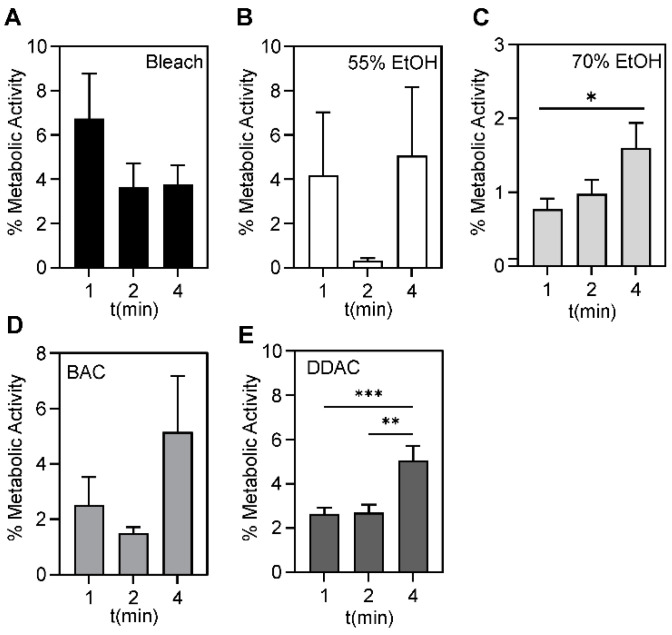
Bacterial biofilm survival following microbicide exposure. No significant differences were seen in the average metabolic activity for all strains after 1, 2, or 4 min of exposure to (**A**) 0.63% bleach and (**B**) 55% ethanol. (**C**) A small, but significant increase was observed at 4 min compared to 1 min exposure to 70% ethanol (*p* = 0.04). Of the two QACs tested, no significant differences were seen in (**D**) 0.5% BAC, and small, but significant differences were observed after (**E**) 4 min compared to 1 min (*p* = 0.0009) and 2 min (*p* = 0.0012) of 0.61% DDAC exposure. The average metabolic activity of all strains is displayed and error bars represent the standard deviation. A *p*-value of ≤0.05 was considered significant. * *p*-value < 0.05, ** *p*-value < 0.01, *** *p*-value < 0.001.

Increasing the contact time did not significantly affect bacterial survival after treatment with 0.63% bleach, 55% ethanol, or 0.50% DDAC (Figure 5, Table S4). After treatment with 70% ethanol, a small, but significant increase in survival was noted after 2 min (*p* = 0.04) and 4 min (*p* = 0.02) compared to 1 min. After treatment with 0.61% BAC, a similar small increase was observed at 2 min (*p* = 0.0009) and 4 min (*p* = 0.001) compared to 1 min (Figure 5D,E and Table S4). It is likely that these small observed increases are not biologically significant.

**Figure 6:**
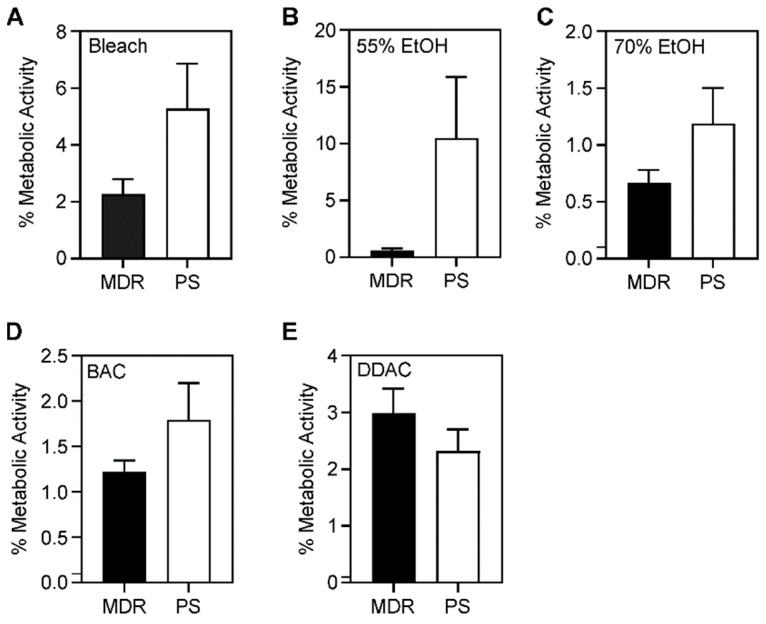
Comparison of survival of bacterial biofilms formed by multidrug-resistant and pan-susceptible strains. Average percentage of metabolic activity of five MDR versus five PS biofilms after (**A**) 4 min of bleach, (**B**) 1 min of 55% ethanol, (**C**) 1 min of 70% ethanol, (**D**) 2 min of 0.5% BAC, and (**E**) 1 min of 0.61% DDAC exposure. The average metabolic activity is displayed; error bars represent standard deviations. A *p*-value of ≤0.05 was considered significant. No significant differences were observed.

**Figure 7:**
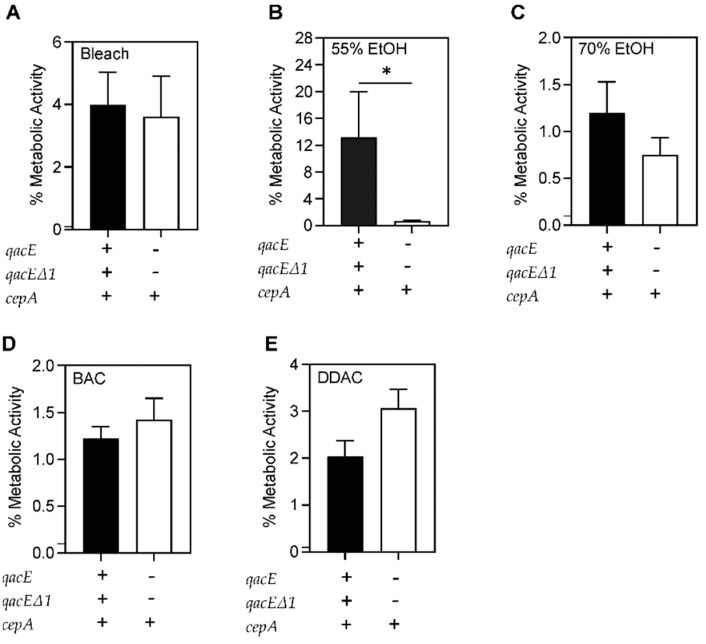
Comparison of survival of bacterial biofilms formed by microbicide-resistant and -susceptible strains. Average percentage of metabolic activity of four microbicide-resistant and six microbicide-sensitive strains. No significant differences were seen between resistant and sensitive strains after (**A**) 4 min of bleach exposure. (**B**) Significantly more microbicide-resistant bacteria survived compared to sensitive strains after 1 min of 55% ethanol exposure (*p* = 0.04). No significant differences between resistant and sensitive bacteria were seen after (**C**) 1 min of 70% ethanol, (**D**) 2 min of 0.5% BAC, and (**E**) 1 min of 0.61% DDAC exposure. The average metabolic activity of is displayed and error bars represent the standard deviation. A *p*-value of ≤0.05 was considered significant. * *p*-value < 0.05.

Bacterial biofilm survival was further evaluated based on the resistance pattern in each microbicide (bleach, 55% and 70% ethanol, BAC, and DDAC) using unpaired *t*-tests. No significant differences were seen between MDR and PS bacterial survival in any of the microbicides after biofilm formation (Figure 6). However, more microbicide-resistant compared to microbicide-sensitive bacteria survived after 55% ethanol treatment (Figure 7B). No significant differences were seen between microbicide-resistant and sensitive isolates in other antiseptics (Figure 7).

## 3. Discussion

Hospital-acquired infections can be detrimental to a patient’s health, and outbreaks can be influenced by a multitude of external factors. From 2012–2018, the number of CRAB infections was downtrending [53]. The hospital rates of carbapenem resistance were approximately 20–35% based on antibiograms, but resistance can vary year to year [54]. Recently, the COVID-19 pandemic resulted in increased CRAB hospital outbreaks [55,56,57], which may be attributable to empiric antibiotic overuse [56]. Given the current crisis of antibiotic resistance, preventing and controlling outbreaks has become more important than ever. Besides practicing antimicrobial stewardship, proper decontamination—a term defined by the Occupational Safety and Health Administration (OSHA) to describe the process by which pathogenic organisms are destroyed either physically or chemically—should be employed. This process can be through cleaning, sanitizing, disinfecting, or sterilizing. Cleaning refers to the removal of pathogens using a detergent or surfactant. Sanitizing refers to reducing the number of pathogens within 30 s. Disinfecting refers to the destruction of pathogens, except for sporulating organisms. Sterilizing is the process by which all pathogens are effectively killed, including those that undergo sporulation [26]. The germicidal formulations used in this study mimic hospital disinfectant wipes used by Cooper University Hospital and are approved by the Environmental Protection Agency (EPA) to be effective against specific MDR *A. baumannii* strains after one a minute exposure of bleach and DDAC, but two minutes for BAC.

The goals of this study were to understand the implications of antibiotic and microbicide-resistance among *A. baumannii* isolates and to assess their potential contribution to hospital outbreaks. We selected disinfecting wipes and skin antiseptics that are routinely used in Cooper University Hospital. We showed that all wipe formulations were effective disinfectants against free-living, planktonic *A. baumannii* strains at 1, 2, and 4 min. Since we tested five different hospital wipes and two skin antiseptics, we compared the efficacy of these different microbicides. We found that povidone, CHG, and the QACs killed 100% of planktonic bacteria. However, following exposure to 0.63% bleach, 55% ethanol, and 70% ethanol at their proper dwell times, a small subpopulation (<0.1%) of bacteria repeatedly survived. For disinfectants to properly destroy pathogens, they must be used at the proper dwell time. The standard dwell times for DDAC against *A. baumannii* is 1 min and 2 min for BAC [46,47]. Bleach is a disinfectant at 1 min, but sterilizes at 4 min, and common hospital practice recommends using a contact time of 4 min [44]. 50% ethanol is generally germicidal after 60 s and 70% ethanol is germicidal after 30 s [45]. Even at an extended dwell time of 4 min, we found that a small subpopulation remained (Figure 1 and Table S2). This suggests that in any population of *A. baumannii*, microbicide-tolerant persisters may exist, even if the strains were not exposed to these agents prior. Bacterial persistence is a commonly observed phenomenon during drug exposure and is believed to be a factor in recurring bacterial infections [58,59]. Recently, bacterial persisters were found in *Escherichia coli* in response to QACs [60]. It is possible that these persisters contribute to the spread of *A. baumannii* infections in hospital settings, as these bacteria survive well on hard surfaces [4,12,20,21,22,23,24,25]. These results were obtained following proper dwell times, so this may become even more significant if improper disinfecting techniques are used.

Antibiotic resistance is selected for when a subpopulation of bacteria survives exposure to a drug, likely by upregulating efflux pumps or obtaining additional resistance genes through plasmid exchange [61]. A similar phenomenon likely exists for microbicide tolerance or resistance. We hypothesized that MDR strains either more easily acquire additional determinants that confer microbicide resistance or contain drug-resistance determinants that confer cross-protection against microbicides. We expected that the MDR strains would be more resistant to some microbicides than PS strains. While we did not find any differences in the percentage of survival between MDR and PS strains during the time-kill assays with bleach (Figure 2), the MDR strains had a higher average MBC. With a higher MBC, this made the MBC/MIC ratio >4, indicating that bleach is not bactericidal against these strains, but is bacteriostatic (Table 2). This was also true for the microbicide-resistant strains. The MDR strains also had an increased MBC to ethanol (Table 3), whereas the microbicide-resistant strains had an increased MBC in isopropanol (Table 4), but both these agents were still bactericidal. The increase in MBC indicates an evolutionary trend and may imply that upon further exposure to the microbicide, it is more likely that they will develop increased tolerance or resistance [41]. These findings highlight the importance of using antiseptics well above the MIC or MBC to ensure proper disinfection [62]. It is also important to note that we only evaluated ten strains; future studies should evaluate more strains to better understand the relationship between antibiotic and microbicidal-resistance genes.

The relationship between microbicidal and drug-resistance is equivocal. Even though some studies identified the presence of microbicidal-resistance genes in MDR pathogens [5,34,36,39], evidence of phenotypic resistance has not been demonstrated in vitro [39,40]. On the other hand, some research has been able to demonstrate such a relationship [42]. It is likely that microbicide tolerance, rather than antibiotic resistance is the significant driver determining bacterial survival in adverse environments [5,32,35,37,42]. We screened our collection for several known microbicidal-resistance genes from the SMR family that included the common efflux pump genes *qacA*, *qacE*, and *qacEΔ1*, and *cepA*. The *qac* resistance genes confer resistance to quaternary ammonium compounds, such as BAC and DDAC [35]. They also can confer resistance to specific antimicrobials, such as *qacE* with sulfonamides [35] and *cepA* with CHG [5,32,37]. Our data does not demonstrate a relationship between the two types of resistance, as microbicidal-resistance gene distribution in our strain collection was not associated with antimicrobial resistance (Table 1). Only one MDR strain in our collection had the three microbicide-resistance genes whereas and all others were PS. It is possible that MDR strains continue to acquire additional microbicidal-resistance genes during the evolution of a prolonged hospital outbreak. Of course, it is also possible that there are additional, uncharacterized resistance mechanisms in our collection. In addition, we do not know which efflux systems are expressed in these strains, if any. While there was no planktonic bacterial survival after QAC, povidone or CHG exposure among microbicide-resistant strains, there was a significant increase in bacterial survival after bleach treatment compared to microbicide-susceptible strains (Figure 3). This suggests that some of these efflux pumps may be expressed and that their expression confers more tolerance to bleach exposure.

As we did not observe differences among free-living MDR and PS bacteria, we thought that differences might emerge following biofilm growth. The bacterial biofilm encases the cells an extracellular matrix that protects cells in vivo from immune defenses and antibiotics, and protects them from environmental insults such as surface disinfectants [63,64]. In the natural environment, the ability to form biofilms is critical for *A. baumannii* survival [65]. We assessed microbicide efficacy against *A. baumannii* following biofilm formation. We found that larger percentages of the population survived, when compared to free-living bacteria. On average, 3.7%, 5.6%, 0.9%, 1.5%, and 2.6% of bacteria survived after exposure to 4 min of bleach, 1 min of 55% ethanol, 1 min of 70% ethanol, 2 min of 0.5% BAC, and 1 min of 0.61% DDAC, respectively (Table S3). Again, increasing the exposure time did not result in an increase in bacterial killing (Table S4). Rather, we observed that longer exposure to 70% ethanol and 0.61% DDAC resulted in statistically significant more bacterial survival compared to the 1-min time point. The small observed increase in survival is likely not biologically relevant. In addition, no differences were observed between MDR and PS strains (Figure 6), however differences were seen between microbicide-resistant and sensitive strains (Figure 7), suggesting that drug resistance is a separate phenomenon from microbicide resistance. This data suggests that the surviving subpopulation is tolerant or resistant to these microbicidal agents. Similar to a prior study which highlighted the connection between biofilm formation and antiseptic resistance [28], our findings of bacterial survival of >0.1% demonstrate that biofilm formation is an innate mechanism of microbicide tolerance. Together, our results suggest the need for a more extensive decontamination process when biofilms might be involved. For instance, a previous study demonstrated that it took up to 10 min to destroy *A. baumannii* biofilms [66]. Furthermore, if there is extensive grime or other visible soil, additional wipes and pre-cleaning with applied friction is recommended for disinfection [44,46,47]. Our findings, along with prior studies suggest that bacterial biofilms may require pre-cleaning prior to disinfection. As it is difficult to know when bacterial biofilms are present, the safest option is to implement a pre-cleaning step with use of friction as part of standard hospital operating procedures.

In sum, a more holistic approach that extends beyond surface decontamination may be needed to combat HAI’s. Surfaces in hospitals and health care facilities serve as reservoirs for pathogens, and hands are usually the vector transferring those microorganisms to patients. Thus, hand hygiene is important in minimizing transfer of pathogens. Cleaning and disinfecting the environment where the patient resides is fundamental to reduce the microorganism burden in patient’s environment. Most, if not all housekeeping surfaces need to be cleaned with soapy water or a detergent disinfectant, according to product specifications, where an emphasis is placed on scheduling and the technique used to clean and disinfect [67,68]. In addition, to focus on the environmental surfaces, there has been heavy reliance on development of strategies to prevent HAIs, often referred to as “bundles” of infection prevention. A method called the “bundle” approach consists of ongoing hospital staff education with regards to maintaining strict hand hygiene, isolation precautions, environmental cleaning, and surveillance of high-risk areas. In 2009, a hospital in Spain assessed the long-term impact of the bundle approach from 1994 to 2003. Results showed that the rates of colonization and infection significantly decreased from 0.82 cases per 100 admissions in 1994, to 0.46 in 1996–1997, to 0.21 in 1998–2003 [69]. Thus, while proper decontamination is important, a multi-disciplinary approach is essential when attempting to combat this problematic pathogen.

## 4. Materials and Methods

### 4.1. Bacterial Strains, Media, and Growth Conditions

Ten *A. baumannii* patient isolates were collected during a routine workup performed by the clinical microbiology lab at Cooper University Hospital, Camden, NJ, USA. The isolates were collected during a hospital outbreak from 2004–2005 and included strains from sporadic cases from 2007–2012. The bacterial isolates were de-identified of all patient information. Since the goals of the project did not include interactions or interventions with living individuals or their private identifiable data, the Cooper Human Research Protection Program determined that this project did not involve human subjects, and as a result, the present research did not require an IRB review. We selected five MDR and five PS strains, as determined by the susceptibility testing during routine workup. Liquid and agar Mueller-Hinton broth (MHB) and Luria broth (LB) were prepared following standard protocols. For time-kill assays with planktonic bacteria, cells were grown overnight in liquid MHB at 37 °C with aeration. For biofilm assays, starter cultures were grown overnight in liquid LB at 37 °C with aeration. Growth was monitored by measuring the optical density at 600 nm (OD_600_) when appropriate.

### 4.2. Time Kill Assays with Planktonic Bacteria

Bacterial strains were grown overnight in MHB at 37 °C with aeration. The following day, 100 μL of stationary phase cells were added to 900 μL of diluted antiseptic in MHB, or MHB alone. The following commonly used hospital disinfectant wipes were recreated as solutions: 55% and 70% ethanol, 0.63% bleach, and two high alcohol QAC’s: (1) 0.61% dodecyl dimethyl ammonium chloride (Millipore Sigma, Burlington, MA, USA) in 27% ethanol and 25% isopropanol, and (2) a combination of 0.25% alkyl dimethyl ethyl benzyl ammonium chloride (Millipore Sigma) and 0.25% alkyl dimethyl benzyl ammonium chloride (Beantown Chemical, Hudson, NH, USA) in 55% isopropanol. Skin antiseptics tested were 2% chlorohexidine gluconate (ThermoFisher Scientific, Waltham, MA, USA), and 10% povidone (ThermoFisher Scientific). The cells were incubated in the presence or absence of microbicides for 1, 2 and 4 min. At each time point, cells were centrifuged, washed twice with phosphate buffered saline (PBS, 10 mM potassium phosphate, pH 7.4, 0.15 M NaCl) and resuspended in 1 mL PBS. Serial dilutions were performed, plated on LB and incubated overnight at 37 °C. Prior to antiseptic treatment, cells were serially diluted and plated to determine initial culture counts. The next day, colony-forming units (CFUs) were counted for each time point, and percent survival determined as antiseptic-resistant CFUs/mL divided by the initial CFUs/mL. Experiments were performed in triplicate if some bacterial survival was noted or in duplicate if there was no bacterial survival (i.e., povidone, CHG, and QAC’s).

### 4.3. Time Kill Assays with Bacterial Biofilms

Bacterial biofilms were grown as previously described [70]. Briefly, overnight cultures were diluted into fresh LB media at a starting OD_600_ of 0.1. 200 μL of these solutions were added to a flat-bottomed 96-well plate and grown for 48 h to allow for biofilm formation. Following incubation, non-adherent bacteria were removed by washing three times with an equal volume of PBS. Four wells were used for each strain. MHB alone served as a sterility control. The other three wells were incubated in the presence of an antiseptic for 1, 2 and 4 min. The antiseptics tested were 0.63% bleach, 55% and 70% ethanol, 0.61% DDAC [46], and 0.50% BAC [47]. The wells were washed three times with 100 μL PBS. To estimate survival of cells in the biofilm, XTT reduction assays were performed as described, with minor modifications [71]. A stock solution of 1 mg/mL XTT (Alfa Aesar, Tweksbury, MA, USA) was made in PBS, which required heating to ~50 °C to fully dissolve the XTT. A 0.4 mM stock solution of menadione (MP Biomedicals) was made in dimethyl-formamide (DMF). Both solutions were filter sterilized with a 0.22 μM filter. To make a working solution, 1.5 mL of the XTT stock solution was mixed with 300 μL of the menadione stock solution. To each well, 200 μL of PBS and 12 μL of the XTT-menadione working solution was added. The plates were wrapped in foil and incubated overnight at 37 °C. The OD_450_ was measured the following day, using a Synergy H1 Microplate reader (Biotek). Prior to analysis, the background was subtracted from each reading. Each experiment was performed three independent times.

### 4.4. Polymerase Chain Reaction

Colony PCR was performed to detect the presence of the known microbicide-resistance genes *qacA*, *qacE*, *qacEΔ1*, and *cepA*. The primers used for amplification are listed in Table 5, and conditions used for the reaction were as previously described [5]. The PCR products were resolved on a 1% TAE agarose gel, and bands visualized with EZ Vision dye (Avantor). All PCR reactions were performed two independent times.

### 4.5. Minimum Inhibitory Concentration (MIC) and minimum Bactericidal Concentration (MBC) Determinations

The MICs to the microbicides were determined by broth microdilution assay, according to standard protocols [72]. The OD_600_ of overnight cultures were determined, and cells diluted to a starting OD_600_ of 0.05. The starting concentration (“X”) for each antiseptic agent is listed in Table 6. Each antiseptic was added at a 2X concentration to the first column of a 96-well plate. Two-fold serial dilutions were performed, and an equal volume of diluted cells was added to each well. The plates were incubated overnight at 37 °C without shaking. The following day, the OD_600_ values were read using a Synergy H1 Microplate reader (Biotek). All MIC determinations were made at least two independent times. To determine MBCs, 25 μL of cells from each well where no growth was observed were plated on LB. Plates were incubated overnight at 37 °C, and following incubation, plates were observed for bacterial growth. The lowest concentration where no growth was observed, was reported as the MBC. All determinations were made at least two independent times.

### 4.6. Statistical Analyses

Statistical analyses were performed using GraphPad Prism 9. The DUOT outlier test was performed on replicates and removed 5% of outliers. If outliers were removed, the experiment was repeated to produce at least three, biological replicates. For statistical comparisons, strains were grouped based on resistance patterns, i.e., MDR vs. PS and presence of multiple microbicide-resistance genes (microbicide-resistant, *cepA*, *qacE*, and *qacEΔ1*) vs. *cepA* alone (microbicide-sensitive), for both planktonic and biofilms conditions. One factor ANOVAs with post-hoc Tukey tests were performed to evaluate whether the type of bacterial resistance affected bacterial survival with varying dwell times or varying types of microbicides at proper dwell time. Unpaired *t*-tests were used to compare MDR and PS strains, and microbicide-resistant and microbicide-sensitive strains for each microbicide. A *p* value ≤ 0.05 was considered statistically significant.

## 5. Conclusions

Our findings highlight that antibiotic resistance may occur by mechanisms other than microbicidal resistance and may be expressed independently in some MDR strains. Microbicide-resistance genes were not necessarily associated with MDR strains, as in our collection, it was more likely that the PS strains contained such resistance genes. The presence of such genes does not mean that they are expressed; however, there was increased survival among planktonic bacteria to some antiseptic agents in the presence of these genes, suggesting that one or more might be expressed. Currently, some hospital policies require isolation precautions only if the pathogen is MDR. Given our findings of microbicide resistance even amongst PS strains, hospitals may want to consider stricter contact precautions, even among infections with PS *A.* *baumannii*. In addition, while the 0.1% surviving subpopulation represents a small number of bacteria in the population, they may still contribute to hospital outbreaks. It is important to note that our study only evaluated 10 strains, and thus, is a work in progress, requiring further study with a larger sample size to properly evaluate the clinical significance of this subpopulation’s role in perpetuating hospital outbreaks. Finally, and perhaps more pertinent to microbicide resistance, when biofilm formation occurred, a noticeable reduction in disinfectant effectiveness was observed. Therefore, the main form of microbicide tolerance may be due to biofilm formation rather than the expression of microbicide-resistance genes. However, these possibilities are not mutually exclusive, and it may be that the combination of specific resistance genes in bacteria that readily form biofilms is the most problematic in terms of their removal from hospital surfaces. This highlights the need for more thorough decontamination processes that include pre-cleaning in addition to disinfection.

## Figures and Tables

**Table 1:** Distribution of known microbicide-resistance genes among *A. baumannii* strains.

Strain	Resistance Pattern	*qacA*	*qacEΔ1*	*qacE*	*cepA*
3	PS	-	-	-	+
4	MDR	-	-	-	+
5	PS	-	-	-	+
9	MDR	-	-	-	+
49	MDR	-	-	-	+
51	MDR	-	-	-	+
54	PS	-	+	+	+
55	MDR	-	+	+	+
56	PS	-	+	+	+
58	PS	-	+	+	+

**Table 2:** Average MICs and MBCs of *A. baumannii* strains following bleach exposure.

	MIC	MBC	MBC/MIC
All strains	0.037	0.098	2.65
MDR	0.035	0.156	4.46
PS	0.039	0.039	1.00
*qacE*, *qacEΔ1*, *cepA*	0.039	0.186	4.77
*cepA*	0.036	0.137	3.81

**Table 3:** Average MICs and MBCs of *A. baumannii* strains following ethanol exposure.

	MIC	MBC	MBC/MIC
All strains	5.94	8.125	1.37
MDR	6.25	10	1.60
PS	5.63	6.25	1.11
*qacE*, *qacEΔ1*, *cepA*	5.47	7.81	1.43
*cepA*	6.25	8.33	1.33

**Table 4:** Average MICs and MBCs of *A. baumannii* strains following isopropanol exposure.

	MIC	MBC	MBC/MIC
All strains	6.25	8.33	1.33
MDR	6.25	7.81	1.25
PS	6.25	8.75	1.40
*qacE*, *qacEΔ1*, *cepA*	6.25	10.94	1.75
*cepA*	6.25	6.25	1.00

**Table 5:** Primers used in this study.

Name	Sequence (5′ → 3′)	Reference
qacE_For	CCCGAATTCATGAAAGGCTGGCTT	[5]
qacE_Rev	TAAGCTTTCACCATGGCGTCGG	[5]
qacΔE1_For	TAGCGAGGGCTTTACTAAGC	[5]
qacΔE1_Rev	ATTCAGAATGCCGAACACCG	[5]
cepA_For	CAACTCCTTCGCCTATCCCG	[5]
cepA_Rev	TCAGGTCAGACCAAACGGCG	[5]
qacA_For	GCTGCATTTATGACAATGTTTG	[39]
qacA_Rev	AATCCCACCTACTAAAGCAG	[39]

**Table 6:** Starting concentrations for MIC assays.

Antiseptic Component	“X” Value
Bleach	5.0%
Ethanol	50%
Isopropanol	50%
Dodecyl dimethyl ammonium chloride	4.88%
Alkyl dimethyl ethyl benzyl ammonium chloride	1.12%
Alkyl dimethyl benzyl ammonium chloride	1.12%
Chlorhexidine gluconate	2.0%

## Data Availability

Not applicable.

## Supplementary Materials

The following supporting information can be downloaded at: https://www.mdpi.com/article/10.3390/antibiotics11050614/s1, Table S1: Percent survival of planktonic bacteria following microbicide exposure at proper contact times, Table S2: Average survival following microbicide exposure of planktonic bacteria, Table S3: Percent survival of bacterial biofilms following microbicide exposure at proper contact times and Table S4: Average survival following microbicide exposure of bacterial biofilms.
