# Supplementary material for: Evaluating the Effectiveness of Hospital Antiseptics on Multidrug-Resistant Acinetobacter baumannii: Understanding the Relationship between Microbicide and Antibiotic Resistance
Source: Antibiotics (Basel). 2022 May 3;11(5):614. doi: 10.3390/antibiotics11050614 (PMC9137960; doi:10.3390/antibiotics11050614)
Supplement: Supplementary file 1 [file antibiotics-11-00614-s001.zip › antibiotics-1680980-supplementary.pdf]

Supplemental Material

Evaluating the Effectiveness of Hospital Antiseptics on Multidrug-Resistant *Acinetobacter baumannii*: Understanding the Relationship between Microbicide and Antimicrobial Resistance

Melanie Betchen, Holly M. Giovinco, Michael Curry, Jackson Luu, Henry Fraimow, Valerie J. Carabetta and Raquel Nahra

Table of Contents

Table S1: Percent survival of planktonic bacteria following microbicide exposure at proper contact times 2

Table S2: Average survival following microbicide exposure of planktonic bacteria 3

Table S3: Percent survival of bacterial biofilms following microbicide exposure at proper contact times 4

Table S4: Average survival following microbicide exposure of bacterial biofilms 5

1

Table S1: Percent survival of planktonic bacteria following microbicide exposure at proper contact times

	**All Strains**	**PS**	**MDR**	***cepA***	***qacE, qacEΔ1, cepA***
0.63% bleach	0.08%	0.07%	0.03%	0.01%	0.10%
55% ethanol	0.06%	0.06%	0.06%	0.05%	0.07%
70% ethanol	0.02%	0.01%	0.02%	0.01%	0.02%
10% Povidone	0%	0%	0%	0%	0%
2.0% CHG	0%	0%	0%	0%	0%
0.5% BAC	0%	0%	0%	0%	0%
0.61% DDAC	0%	0%	0%	0%	0%

CHG: chlorhexidine gluconate. 0.5% BAC: 0.25% alkyl dimethyl ethyl benzyl ammonium chloride, 0.25% alkyl dimethyl benzyl ammonium chloride in 55% isopropanol. 0.61% DDAC: 0.61% dodecyl dimethyl ammonium chloride in 27% ethanol and 25% isopropanol. PS: pan-susceptible. MDR: multidrug resistant.

Table S2: Average survival following microbicide exposure of planktonic bacteria

	1 minute	2 minutes	4 minutes
0.63% bleach	0.08%	0.05%	0.05%
55% ethanol	0.06%	0.06%	0.06%
70% ethanol	0.02%	0.02%	0.03%
10% Povidone	0%	0%	0%
2.0% CHG	0%	0%	0%
0.5% BAC	0%	0%	0%
0.61% DDAC	0%	0%	0%

CHG: chlorhexidine gluconate. 0.5% BAC: 0.25% alkyl dimethyl ethyl benzyl ammonium chloride, 0.25% alkyl dimethyl benzyl ammonium chloride in 55% isopropanol. 0.61% DDAC: 0.61% dodecyl dimethyl ammonium chloride in 27% ethanol and 25% isopropanol.

Table S3: Percent survival of bacterial biofilms following microbicide exposure at proper contact times

	**All Strains**	**PS**	**MDR**	***cepA***	***qacE, qacEΔ1, cepA***
0.63% bleach	3.77%	5.3%	2.3%	0.04%	0.036%
55% ethanol	5.97%	10.5%	0.6%	0.007%	0.13%
70% ethanol	0.90%	0.029%	0.007%	0.008%	0.012%
0.5% BAC	1.51%	0.018%	0.012%	0.014%	0.012%
0.61% DDAC	3.0%	0.023%	0.03%	0.031%	0.02%

CHG: chlorhexidine gluconate. 0.5% BAC: 0.25% alkyl dimethyl ethyl benzyl ammonium chloride, 0.25% alkyl dimethyl benzyl ammonium chloride in 55% isopropanol. 0.61% DDAC: 0.61% dodecyl dimethyl ammonium chloride in 27% ethanol and 25% isopropanol. PS: pan-susceptible. MDR: multidrug resistant.

Table S4: Average survival following microbicide exposure of bacterial biofilms

Antiseptic	1 minute	2 minutes	4 minutes
0.63% bleach	6.7%	3.6%	3.7%
55% ethanol	5.8%	0.6%	5.4%
70% ethanol	0.9%	1.1%	2.4%
0.5% BAC	2.5%	1.5%	5.1%
0.61% DDAC	2.7%	2.7%	5.1%

CHG: chlorhexidine gluconate. 0.5% BAC: 0.25% alkyl dimethyl ethyl benzyl ammonium chloride, 0.25% alkyl dimethyl benzyl ammonium chloride in 55% isopropanol. 0.61% DDAC: 0.61% dodecyl dimethyl ammonium chloride in 27% ethanol and 25% isopropanol.
